# MicroRNAs as Biomarkers for Liver Disease and Hepatocellular Carcinoma

**DOI:** 10.3390/ijms17030280

**Published:** 2016-02-24

**Authors:** C. Nelson Hayes, Kazuaki Chayama

**Affiliations:** 1 Department of Gastroenterology and Metabolism, Applied Life Sciences, Institute of Biomedical and Health Sciences, Hiroshima University, 1-2-3 Kasumi, Minami-ku, Hiroshima 734-8551, Japan; nelsonhayes@hiroshima-u.ac.jp; 2 Liver Research Project Center, Hiroshima University, Hiroshima 734-8551, Japan; 3 Laboratory for Digestive Diseases, Center for Genomic Medicine, RIKEN, Hiroshima 734-8551, Japan

**Keywords:** microRNA, non-coding RNA, viral hepatitis, inflammation, occult HBV, hepatocellular carcinoma, fibrosis, HBe antigen, α-fetoprotein, biomarker

## Abstract

Serum levels of liver enzymes, such as alanine transaminase, aspartate transaminase, and α-fetoprotein, provide insight into liver function and are used during treatment of liver disease, but such information is limited. In the case of hepatocellular carcinoma (HCC), which is often not detected until an advanced stage, more sensitive biomarkers may help to achieve earlier detection. Serum also contains microRNAs, a class of small non-coding RNAs that play an important role in regulating gene expression. miR-122 is specific to the liver and correlates strongly with liver enzyme levels and necroinflammatory activity, and other microRNAs are correlated with the degree of fibrosis. miR-122 has also been found to be required for hepatitis C virus (HCV) infection, whereas other microRNAs have been shown to play antiviral roles. miR-125a-5p and miR-1231 have been shown to directly target hepatitis B virus (HBV) transcripts, and others are up- or down-regulated in infected individuals. MicroRNA profiles also differ in the case of HBV and HCV infection as well as between HBeAg-positive and negative patients, and in patients with occult *versus* active HBV infection. In such patients, monitoring of changes in microRNA profiles might provide earlier warning of neoplastic changes preceding HCC.

## 1. Introduction

Hepatocellular carcinoma (HCC) accounts for 90% of primary liver cancers and is the fifth most common cancer worldwide, as well as the third most common cause of cancer-related death [1,2]. Progression of chronic hepatitis to cirrhosis or HCC may occur slowly over several decades, but by the time the cancer is detected, prospects for successful treatment are often poor. Viral hepatitis is responsible for most cases of HCC, with a worldwide incidence of about 54% for hepatitis B virus (HBV) infection and 31% for hepatitis C virus (HCV) [3]. Direct acting antiviral (DAA) therapy, with agents such as sofosbuvir and ledipasvir, is expected to substantially reduce mortality and morbidity among the 185 million people throughout the world chronically infected with HCV, although, the long-term risk of HCC remains high in cirrhotic patients even after achieving sustained virological response (SVR) [4]. No comparable success has been achieved for the 360 million people with chronic HBV infection. Although an effective vaccine has been available since 1986 and most adult infections are successfully resolved during the acute phase, chronic HBV infection remains a serious public health threat requiring long term antiviral therapy with a low probability of complete clearance of the virus due to the long-term stability of viral covalently closed circular DNA (cccDNA) in the nucleus. In these patients, gradual changes and deterioration in liver function must be monitored over long periods. While serum levels of liver enzymes, such as alanine transaminase (ALT), aspartate transaminase (AST), and α-fetoprotein (AFP), provide insight into liver function, enzyme levels convey limited information, and HCC is often not detected until an advanced stage. Therefore, more sensitive biomarkers may help to achieve earlier detection in patients with chronic hepatitis, and serum microRNAs represent a promising approach to monitoring liver function.

## 2. MicroRNAs

MicroRNAs are short, non-coding RNAs consisting of 18–25 nucleotides that are partially complementary to regulatory regions in the 3′ or, less commonly, in the 5′ untranslated region (UTR) of target messenger RNAs. MicroRNA binding suppresses translation of target mRNAs or promotes mRNA degradation, providing a rapid and sensitive mechanism to fine tune gene expression. MicroRNAs serve as guides to position the RNA-induced silencing complex (RISC), a molecular scaffold that facilitates interaction of a microRNA with its target sequence and mediates the inhibitory effect on gene expression [5]. MicroRNAs form complex post-transcriptional regulatory networks that regulate numerous cellular processes [6]. Although not discovered until 1993, microRNAs are now known to affect the expression of at least 30% of human genes, making them the most abundant regulators of gene expression [7]. Around 60% of messenger RNAs (mRNAs) contain a predicted binding site in the 3′ UTR, and many mRNAs contain multiple potential binding sites. However, relatively few of the computationally-predicted microRNA–mRNA interactions have been experimentally validated. 2588 mature human microRNAs and 1881 precursors are currently registered in the miRBase database (Release 21; June 2014). A single microRNA is likely to regulate multiple genes, and conversely a single gene might be regulated by multiple microRNAs. MicroRNA expression may also be tissue- or organ-specific. Since even a small change in microRNA expression may affect expression of hundreds of target genes and significantly alter the transcriptome [8], disruption of microRNA regulatory networks has been implicated in a number of diseases [9,10,11]. MicroRNA involvement in cancer was first reported in 2002 for its role in leukemia [12]. While the predictive utility might vary among individual microRNAs, expression profiles involving multiple microRNAs are expected to play a useful role in tumor classification, diagnosis, and prognosis.

## 3. Production of MicroRNAs

**Figure 1:**
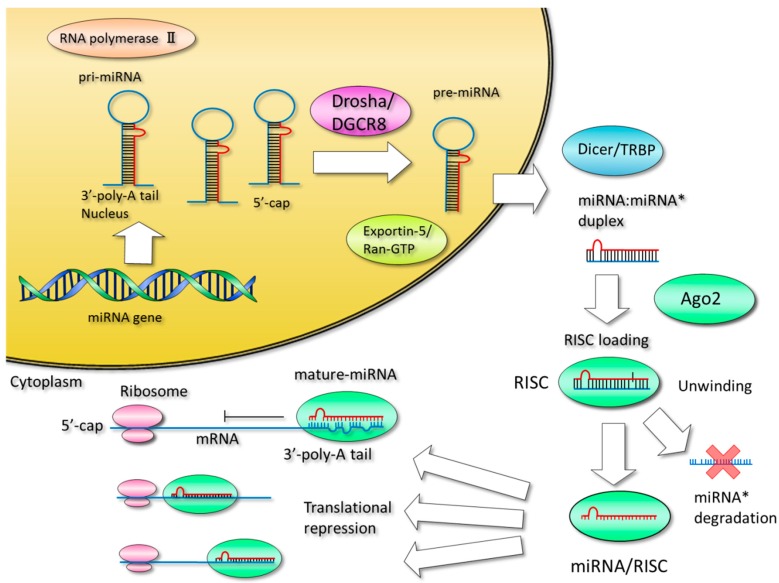
MicroRNA synthesis and function. MicroRNA genes are transcribed by RNA polymerase II to produce primary microRNAs (pri-microRNAs) and cleaved by Drosha/DGCR8 into pre-microRNAs. Pre-microRNAs are exported from the nucleus by exportin-5 and Ran-GTP, and then the hairpin is cleaved by Dicer/TRBP in the cytoplasm. The microRNA duplex is unwound and one strand is complexed with argonuate 2 (AGO2) to form the RNA-induced silencing complex (RISC), and the unused passenger strand (miRNA*) is degraded. The microRNA guides the RISC to a partially complementary target region in the 3′ untranslated regions of one or more genes, resulting in translational repression or target cleavage. Distinct proteins or protein complexes are depicted as colored ovals. Red and blue lines indicate RNA.

MicroRNA genes, often located in the introns of protein-coding genes, are transcribed by RNA polymerase II into long, capped, polyadenylated pri-microRNAs and subsequently processed by Drosha into pre-microRNAs [13] (Figure 1). Pre-microRNA hairpins are exported from the nucleus by exportin-5 and processed into double-stranded mature microRNAs by Dicer. When the microRNA has been incorporated into the RISC complex, the unused complementary strand is degraded. The microRNA serves as a guide to orient the RISC complex in position at regulatory sequences in target genes. While argonaute 2 (AGO2) can directly cleave messenger RNA, particularly in the case of perfect complementarity, argonaute proteins normally facilitate translational inhibition by reducing RNA stability through uncapping and de-adenylation.

## 4. Serum MicroRNAs

Enzymes such as ALT and γ-glutamyl transpeptidase (GGT) are considered liver-specific (although ALT may be elevated with kidney or muscle damage), but other enzymes commonly used in liver function tests such as AST and alkaline phosphatase are also expressed in muscle and bone, respectively, limiting their specificity and requiring more complex interpretation. Conversely, many microRNAs are expressed in a tissue- or organ-specific manner, suggesting that microRNA biomarkers are more likely to have high specificity. The presence of microRNAs in serum and their potential use as biomarkers was first reported by Lawrie *et al.* [14] in 2008 with regard to their role in diffuse large B-cell lymphoma, and the idea has since been pursued in a number of studies. Analysis of differential microRNA expression in liver tissues has identified microRNAs associated with different stages of liver disease, as well as specific microRNA subsets associated with HCV *versus* HBV infection [15,16,17,18,19]. However, direct measurement of tissue microRNAs is intrusive and inconvenient as a biomarker. On the other hand, measurement of serum microRNAs is much less intrusive, and microRNA levels in the liver have been found to be correlated with serum levels for a number of microRNAs [20,21]. MicroRNAs from the liver may enter the serum passively through apoptosis and necrosis or actively through secretion of exosomes and viral particles [22]. Therefore, microRNA levels in the serum might provide a way to estimate microRNA activity in the liver. Comparison of serum microRNA levels before and after tumor resection suggests that circulating microRNAs such as miR-15b, miR-21, miR-130b, and miR-183 originate in tumor cells [23]. Fortunately, microRNA is detectable and relatively stable in serum, plasma, urine, saliva, and cerebrospinal fluid [24], and frozen samples can be stored without substantial degradation. Free RNA is quickly degraded by RNases and, typically, has a short half-life. Conversely, mature microRNAs are much more stable and are normally complexed with AGO2 or other argonaute proteins [5]. Circulating microRNAs may exist as vesicle-free ribonucleoprotein complexes or may be transported within HBV surface antigen (HBsAg) particles or contained within exosomes/microvesicles [5,22,25], although serum microRNAs are typically found in exosomes [26].

## 5. Exosomes

Exosomes are ubiquitous cell-derived vesicles ranging from 30 to 100 nm that have been shown to affect gene expression in recipient cells. Exosomes contain characteristic RNA transcripts, including microRNAs, transfer RNAs, and other types of non-coding RNAs [27], which may vary by cell type but do not necessarily mirror the RNA profile of the parent cell due to selective sorting and response to cellular conditions [27]. miR-99a, miR-128, miR-124, miR-22, and miR-99b account for 49% of identified exosome-associated microRNAs [27]. Hepatocyte-derived exosomes are enriched for gene products involved in lipoprotein metabolism and xenobiotic processing and might, therefore, prove useful as a diagnostic tool by reflecting hepatic changes linked to disease [28]. Although interferon normally acts on cells directly by binding to receptors and triggering expression of numerous interferon-stimulated genes sharing a common response element, viruses, such as HBV and HCV, frequently interfere with interferon signaling and suppress intracellular innate immune responses. An alternative antiviral mechanism was recently described in which interferon stimulates release of exosomes that contain antiviral products, which are then internalized by HBV-infected hepatocytes, bypassing viral interference [29]. The role of exosomes in disease pathogenesis and cancer metastasis has yet to be fully elucidated.

## 6. Measurement of Serum MicroRNAs

To serve as a reliable biomarker, standardized methods for serum microRNA measurement are needed. Many methods exist for isolating RNA from serum but accurate measurement of serum microRNAs is challenging due to the low quantity, short length, and high sequence variability of microRNAs. Initially, RNA is extracted, and the small RNA fraction is enriched. Bulk measurement of microRNA expression can be performed using microarray analysis or next generation RNA sequencing, but validation and absolute quantification of individual microRNAs is typically performed using quantitative real-time polymerase chain reaction [30]. However, microRNA analysis requires additional quality control and normalization steps that remain unstandardized [31]. Selection of an appropriate internal control is difficult due to high variability of small RNAs such as RNU4, RNU6b, and RNU48 among healthy controls [32] or confounding with disease state, such as down-regulation of RNU6b during fibrosis [33]. Similarly microRNAs such miR-15b and miR-16 are sensitive to hemolysis [34]. While the choice of internal control may depend on the nature of the study, miR-24, miR-126, and miR-484 have been suggested for use in normalization [32]. Differentiating among variable-length isomiRs and closely-related microRNAs in the same family as well as between mature microRNAs and pre-/pri-microRNAs poses an additional challenge. In some cases, the status of a non-coding RNA as a microRNA has been called into question. While miR-720 has recently been reported to target the oncogene twist family BHLH transcription factor 1 (TWIST1) involved in tumor metastasis in breast cancer [35], the microRNA entry for miR-720 was removed from miRBase after Schopman *et al.* [36] proposed that the microRNA was actually a mis-annotated tRNA fragment. Examination of miRBase using next generation sequencing data has revealed numerous other such potential conflicts, frequently involving mis-annotation of small nucleolar RNAs (snoRNAs) [37]. Although these RNAs are not microRNAs, recent studies have shown that tRNA fragments and other small non-coding RNA species may serve secondary regulatory or signaling functions and that their presence in serum may vary depending on physiological state [38,39]. Selitsky *et al.* [40] showed that tRNA fragments are more abundant than microRNAs in liver tissue and were significantly elevated in patients with chronic HBV or HCV compared to healthy subjects, suggesting a potential role for other types of non-coding RNA for use as biomarkers. While these will likely remain open issues until the non-coding RNA transcriptome is more fully characterized, the methodology for serum microRNA measurement should standardize once effective microRNA panels have been validated and interest in their use in clinical practice increases in the same way that protocols for performing and reporting genome-wide association studies (GWAS) have become more standardized.

## 7. Baseline MicroRNA Expression in the Liver

Baseline microRNA levels in hepatocytes and liver tissue have been established using deep sequencing methods [41,42,43]. While miR-122 accounts for 70% of the total microRNA in the liver [44], a set of 9 microRNAs including miR-192, miR-199a/b, miR-101, miR-99a, and let-7a/b/c/f accounts for 88% of total microRNA [42]. Genome-wide expression profiling identified a total of 277 microRNAs expressed in the liver, 166 of which were expressed in all samples, including miR-16, miR-27b, miR-30d, miR-126, and several members of the let-7 family [43]. Baseline miR-122 expression is also strongly affected by single nucleotide polymorphisms (SNPs) (rs2999200 and rs6551952), and other SNPs have been shown to affect expression of miR-938, miR-200c, and miR-10b [43]. Some microRNAs show age-dependent differences in expression. 114 microRNAs were up-regulated and 72 were down-regulated in fetal *versus* pediatric liver tissues, whereas only two microRNAs were up-regulated and three were down-regulated in pediatric *versus* adult tissue. Sex-specific differences in microRNA expression have also been observed [45]. miR-29a and miR-29b are induced by estrogen and up-regulated in females [46]. These microRNAs suppress collagen deposition in the extracellular matrix, potentially providing better protection against fibrosis in females. Consideration of factors affecting baseline microRNA expression in the liver may be important in the interpretation of microRNA biomarkers. Validation studies should consider stratifying by age and/or sex and might consider genotyping SNPs of large effect.

## 8. The Role of MicroRNAs in Liver Injury and Disease

MicroRNAs are involved in regulation of numerous metabolic pathways, and changes in serum microRNA profiles might reflect underlying liver injury or inflammation.

### 8.1. Autoimmune Liver Diseases

Autoimmune hepatitis (AIH) and primary biliary cholangitis (PBC) are chronic autoimmune diseases characterized by immune-directed damage to hepatocytes and cholangiocytes, respectively. Diagnosis of autoimmune liver diseases requires differential diagnosis involving clinical and histological findings and exclusion of other factors. A recent study by Migita *et al.* [47] found elevated serum levels of miR-21 miR-122 in patients with AIH compared to heathy controls or patients with HCV, but levels of these microRNAs decreased in patients with cirrhosis. Tan *et al.* [48] showed that a panel including miR-122-5p, miR-141-3p, and miR-26b-5p was diagnostic for PBC with higher sensitivity and specificity than traditional biomarkers, such as alkaline phosphatase (ALP) and antinuclear antibody (ANA). Ideally, microRNA panels such as these will lead to earlier and more accurate diagnosis in patients with autoimmune diseases.

### 8.2. Drug-Induced Liver Injury

Drug-induced liver injury (DILI) results from drug overdose or the hepatotoxic effects of a drug metabolite. Early diagnosis of DILI is important, especially in the case of idiopathic DILI where the underlying cause is difficult to determine, but standard serum biomarkers such as ALT, AST, bilirubin, and alkaline phosphatase have poor sensitivity and specificity, and more accurate biomarkers are needed [49]. In mouse models of acetaminophen-induced liver injury, miR-122 was found to be up-regulated in liver tissue, and miR-122, miR-192, miR-155, miR-125b, and miR-146a were up-regulated in serum or plasma [16,50,51]. However, Wang *et al.* [51] reported an inverse correlation between microRNAs levels in the liver *versus* the plasma, in which elevated plasma levels corresponded with lower levels in liver tissue, and vice versa. Fukushima *et al.* [52] reported that miR-298 and miR-370 were down-regulated in rats within six hours after exposure to acetaminophen or carbon tetrachloride and showed that altered microRNA expression levels were associated with mitochondrial damage. While the number of human microRNA studies of DILI is limited, Starkey Lewis *et al.* [53] found that patients who experienced acetaminophen-related DILI had higher plasma levels of miR-122 and miR-192, and Ding *et al.* [54] found that serum miR-122 levels were up-regulated in subjects exposed to paraquat, a toxic herbicide.

### 8.3. Inflammatory Liver Damage

In contrast, a different pattern was observed for inflammatory liver damage resulting from alcoholic liver disease (ALD) and Toll-like receptor (TLR) activation [50]. miR-122 and miR-155 were up-regulated in serum of alcohol-fed mice, and miR-122, miR-155, and miR-146a were up-regulated following inflammation induced by TLR4/TLR9 ligand administration. Bala *et al.* [50] reported that circulating microRNAs in the exosome-rich fraction *versus* the protein-rich fraction could discriminate between liver injury and inflammation.

### 8.4. Alcoholic Liver Disease

Aside from the liver, alcohol abuse damages multiple organs, including the pancreas, intestinal epithelium, heart, brain, and muscle tissue, but microRNA dysregulation is thought to play a key role in alcoholic liver disease. Mouse models of ALD have shown increased tissue expression of miR-320, miR-486, miR-705, and miR-1224, and decreased expression of miR-27b, miR-214, miR-199a-3p, miR-182, miR-183, miR-200a, and miR-322 [55]. Rat models have shown elevated serum levels of miR-122 and miR-155 in exosomes following alcohol consumption [50]. Due to the systemic effects of alcohol, however, circulating microRNA biomarkers might not originate exclusively from the liver [31]. Chen *et al.* [56] found up- or down-regulation of 32 serum microRNAs in rats, but expression levels between the liver and serum were correlated only for miR-185, miR-199a-3p, miR-214, and miR-490.

### 8.5. Non-Alcoholic Fatty Liver Disease

Excess fat accumulation in the liver also induces inflammation and can result in non-alcoholic fatty liver disease (NAFLD) or non-alcoholic steatohepatitis (NASH). NAFLD is becoming increasingly common, affecting up to 40% of the population, but because proper diagnosis requires a biopsy, incidence is likely to be under-reported and, therefore, less invasive biomarkers are needed for earlier detection [57,58]. In mouse models of obesity, miR-122, miR-34a, miR-31, miR-103, miR-107, miR-194, miR-334-5p, miR-221, and miR-200a were up-regulated, and miR-29c, miR-451, and miR-21 were down-regulated in *ob*/*ob* mice [59,60]. miR-146, miR-152, and miR-200 were up-regulated in rats fed a high-fat diet [61]. In human studies, miR-21 was found to be up-regulated in patients with steatohepatitis [62], and 23 microRNAs, including miR-122, were up-regulated and 23 were down-regulated in patients with NASH, with predicted effects on apoptosis, inflammation, oxidative stress, and lipid metabolism [62]. In serum, levels of miR-122, miR-34a, and miR-16 were up-regulated in patients with NAFLD compared to healthy controls [63]. Using global microRNA profiling, Pirola *et al.* [64] analyzed 84 serum microRNAs and developed a profile based on up-regulation of miR-122, miR-192, miR-19a, miR-19b, miR-125b, and miR-375. A similar study by Tan *et al.* [48] using Illumina sequencing identified a diagnostic panel of microRNAs associated with NAFLD that included miR-122-5p, miR-1290, miR-27b-3p, and miR-192-5p.

### 8.6. Fibrosis

Liver fibrosis is a consequence of repeated cycles of liver damage and repair, resulting in excessive deposition of extracellular matrix proteins [65,66]. While various factors, such as inflammation, oxidative stress, and apoptosis can activate hepatic stellate cells, fibrosis underlies most types of chronic liver disease and is a precursor to cirrhosis and HCC [67]. Within the liver, several microRNAs including miR-21, miR-221/222, and miR-181b promote liver fibrosis through the TGF-β or NF-κB pathways, whereas miR-29b, miR-101, miR-122, and miR-214-3p prevent fibrosis by inhibiting collagen synthesis or suppressing activation of the TGF-β pathway [67]. Serum miR-34a was found to be up-regulated in patients with fibrosis in a stage-dependent manner [63], and miR-571 and miR-513-3p were elevated in patients with cirrhosis [46]. miR-29a was down-regulated in patients with fibrosis in an inversely stage-dependent manner [68]. Due to the central role of fibrosis in liver disease, better understanding of microRNA regulation of inflammation and hepatic stellate cell activation might lead to new therapeutic approaches. Along these lines, anti-fibrotic microRNA therapy involving activation of miR-29 or miR-101 or inhibition of miR-21 has been considered [65].

## 9. The Role of MicroRNAs in Viral Hepatitis

MicroRNAs play important roles in HBV and HCV infection both through regulation of host genes as well as by direct targeting of viral transcripts. Both viruses also disrupt gene expression in the host cell, including microRNA regulatory networks, in order to establish a more permissive environment for viral replication [69]. Several microRNAs might also serve a therapeutic role.

### 9.1. Hepatitis B Virus

Zhang *et al.* [70] used antisense screening in HepG2 to identify host microRNAs involved in HBV replication and found that miR-199a-3p suppressed HBV replication by directly binding to the S protein coding region, whereas miR-210 inhibited replication by binding to the pre-S1 region. Potenza *et al.* [71] similarly found that miR-125a-5p inhibits translation of the S transcript. Additional microRNAs, including let-7, miR-196b, miR-433, miR-511, miR-205, and miR-345 were predicted to recognize targets in the HBV genome [72]. MiR-1231 has high homology with the core and X regions of the HBV genome and is predicted to hybridize with this region [73]. Overexpression of miR-1231 significantly suppressed HBV replication but did not affect expression of interferon-stimulated genes [73]. The highly-expressed liver microRNA miR-122 was found to directly target the viral genome in the region of the HBV polymerase overlapping the 3′ UTR of the core protein [74]. This key microRNA strongly suppresses HBV replication both through direct binding to HBV RNA, as well as indirectly through cyclin G1-modulated p53 activity [74,75,76,77,78]. Other microRNAs that affect HBV replication indirectly through regulation of host proteins include miR-99a, which acts as a tumor suppressor that targets insulin-like growth factor 1 receptor (IGF-1R) and induces cell cycle arrest [27,79]. MiR-99a also suppresses activity of nuclear factor κB (NF-κB), a transcription factor associated with inflammation and tumorigenesis [80]. miR-22 acts as a tumor suppressor [27] and induces cellular senescence by regulating cyclin-dependent kinase inhibitor 1A (CDKN1A), cyclin-dependent kinase 6 (CDK6), sirtuin 1 (SIRT1), and specificity protein 1 (Sp1) [81,82]. miR-22 has been shown to be down-regulated in HBV-related HCC [82]. MiR-141 down-regulates peroxisome proliferator-activated receptor α (PPARα), a liver-enriched nuclear receptor required for efficient transcription of the HBV genome [83]. Conversely, other microRNAs promote HBV replication. miR-1 increases expression of farnesoid X receptor α (FXRα), another nuclear receptor involved in HBV transcription [84]. miR-501 down-regulates hepatitis B virus X interacting protein (HBXIP), which acts as an HBV inhibitor [85]. While several viruses encode their own microRNAs, no virally-encoded microRNAs have yet been confirmed for RNA although, interestingly, Jin *et al.* [86] identified a potential pre-microRNA sequence present in the HBV genome for which the only predicted target was the HBV genome itself.

A number of studies have shown characteristic serum microRNA profiles in patients with chronic HBV infection compared to healthy control subjects. Using Solexa screening followed by validation with quantitative reverse transcription PCR, Li *et al.* [87] identified a set of 13 microRNAs that were up-regulated at least 3-fold in serum of patients with HBV infection, including miR-375, miR-92a, miR-10a, miR-223, miR-423, miR-23b/a, miR-342-3p, miR-99a, miR-122a, miR-125b, miR-150, and let-7c. This set was also able to discriminate between HBV and HCV cases and HBV and HBV-HCC cases. Serum miR-122 levels have been shown to correlate with ALT levels as well as serum levels of miR-22, HBV DNA, and HBsAg [50,88]. Other studies have reported up-regulation of miR-99a and miR-125b in HBV-infected patients [20,89,90], and serum microRNA levels have been shown to be up-regulated in HBeAg-positive patients compared HBeAg-negative patients [89].

The relationship between microRNAs and HBV is complex. HBV down-regulates expression of Drosha, part of the microprocessor complex responsible for converting pri-microRNA into pre-microRNA hairpins during the first stage of microRNA processing. Down-regulation of Drosha might, therefore, have a globally suppressive effect on microRNA expression levels [91]. Novellino *et al.* [22] demonstrated through immunoprecipitation that microRNAs are contained within HBsAg subviral particles, which are produced in excess in infected hepatocytes. As several microRNAs are known to target HBV, knockdown of AGO2 might be expected to relieve inhibition of HBV replication. Instead, however, HBV DNA and HBs antigen levels decreased following siRNA-mediated AGO2 knockdown [20]. HBcAg and AGO2 were found to physically interact and co-localize in the endoplasmic reticulum, and HBs and AGO2 were found to co-localize in several subcellular compartments. These results suggest that AGO2 might play a role in the HBV life cycle.

### 9.2. Hepatitis C Virus

HCV infection disrupts microRNA regulation of multiple pathways, including immune response, antigen presentation, cell cycle, proteasome, and lipid metabolism [19]. At least nine gene targets (peroxisome proliferator-activated receptor γ (PPARG), signal transducer and activator of transcription 3 (STAT3), interferon regulatory factor 1 (IRF1), insulin-like growth factor 1 receptor (IGF1R), fibronectin 1 (FN1), stearoyl-CoA desaturase (SCD), and cAMP responsive element binding protein 1 (CREB1)), regulated by at least 11 microRNAs (miR-130a/b, miR-200, miR34a, miR-23b, miR-24, miR-146a, miR-381, miR-25*, miR-200a, and miR-371-5p) were altered as a result of HCV infection [92]. Similarly, Peng *et al.* [69] used a graph theoretical approach to identify 38 microRNA-mRNA regulatory modules associated with HCV infection. The host immune response employs microRNAs both to control expression of target genes as well as to directly target the HCV genome [8,69,93]. HCV replication is highly dependent on miR-122, which binds to and helps to stabilize the 5′ UTR (S1 and S2) and the 3′ UTR (S3) of the HCV RNA genome [94,95]. Sequestration of this microRNA dramatically reduces HCV RNA abundance [94,95]. Miravirsen, a 15 nucleotide locked nucleic acid (LNA) that binds to miR-122 and inhibits its function is undergoing clinical trials as an anti-HCV therapy [96]. MiR-141, miR-192, miR-215, and miR-491 also promote HCV replication. miR-141 promotes HCV replication by down-regulating tumor suppressor deleted in liver cancer 1 (DLC-1) [96], and miR-491 facilitates HCV entry through the PI3 kinase/Akt pathway [97]. While these microRNAs are required or exploited by HCV for replication, other microRNAs including miR-199a, miR-196, miR-29, let-7b, miR-130a, and miR-27a show antiviral activity against HCV [98,99,100,101,102,103]. Murakami *et al.* [98] demonstrated that miR-199a* inhibits viral replication by binding to a complementary target sequence within domain II of the internal ribosomal entry site (IRES). While interferon is normally associated with up-regulation of interferon-stimulated genes, Pederson *et al.* [93] showed that interferon-β induces expression of several microRNAs with antiviral activity against HCV, including miR-196, miR296, miR-351, miR-431, and miR-448, of which miR-196 and miR-448 may directly target HCV RNA.

## 10. Hepatocellular Carcinoma

In addition to being the most common form of primary liver cancer, HCC is the fifth most common cancer worldwide and the third most common cause of cancer-related death, responsible for 500,000 deaths per year [1]. Incidence is highest in East Asia and other regions where hepatitis B virus is endemic. Multiple risk factors have been implicated, including chronic HBV or HCV infection, gender, age, alcohol, and aflatoxin B exposure. Given these diverse etiologies, HCC is, accordingly, a complex disease characterized by stepwise accumulation of genetic and epigenetic changes, including mutations, translocations, amplifications or deletions, chromatin remodeling, changes in DNA methylation, and dysregulation of non-coding RNAs [104,105,106].

The five year survival rate is less than 10%, due in part to resistance to chemotherapy and a high recurrence rate. Resection offers the best chance of curative therapy for HCC, but only 10% of patients are eligible at the time of detection. However, the survival rate following resection approaches 70% when the tumor is single and smaller than 2 cm. Therefore, earlier diagnosis is essential in order to improve prognosis. AFP (>400 ng/mL) is the most commonly used biomarker for HCC, but has only modest sensitivity and accuracy and fails to detect HCC in half of patients. Another marker, des-γ-carboxy prothrombin (DCP), also known as prothrombin induced by vitamin K absence-II (PIVKA-II), is an abnormal, nonfunctional form of prothrombin that does not undergo N-terminal carboxylation prior to secretion [107]. The carboxylase responsible is frequently lacking in HCC cells. In combination with AFP and AFP-L3, these biomarkers are predictive of progression of HCC in patients with chronic HBV or HCV, particularly with respect to portal vein invasion or intrahepatic metastasis [107,108]. However, DCP elevation may be due to other causes, and normal DCP does not preclude HCC. Therefore, additional biomarkers are needed, especially those associated with early changes to improve prognosis.

MicroRNA dysregulation is involved in all stages of hepatocarcinogenesis, and microRNA profiles have the potential to discriminate patients with HCC from healthy subjects as well as those with other liver diseases [27,109]. MicroRNA profiles differ between benign and malignant tissue and may vary by malignant subtype [7]. Detectable in liver tumor tissue, serum, plasma, and urine, microRNAs might also provide a minimally invasive way to monitor response to therapy and establish prognosis. A number of studies have reported microRNAs associated with HCC. miR-17-92, miR-21, miR-221, miR-222, miR-224 are frequently up-regulated in HCC tumors [109,110], whereas let-7, miR-200, miR-29, miR-122, miR-123, miR-199a, miR-199b, are often down-regulated [7,109,111]. While miR-122 is down-regulated in primary HCC tumors, serum miR-122 is up-regulated in patients with HCC [112], possibly due to miR-122 release from tumors into circulation. miR-199 is highly expressed in normal liver tissue but is consistently down-regulated in HCC [42]. Since miR-199a/b-3p suppresses HCC in part by inhibiting the p21-activated kinase 4 (PAK4)/Raf/MEK/ERL pathway, down-regulation of miR-199a/b is associated with poor survival. Similarly, miR-99a down-regulation is associated with poor prognosis [79]. Conversely, miR-224 has been reported to be up-regulated in HCC [113] and was recently shown to reflect tumor stage and liver function, with elevated levels associated with reduced survival [114].

Etiology-related differences in microRNA expression may complicate efforts to develop effective biomarker panels due to geographic differences in the underlying causes of HCC. In a recent study, microRNA expression profiling of liver tissue was used to identify dysregulated HBV- or HCV-HCC-associated microRNAs [115]. Of the 40 differentially expressed microRNAs, 10 were strongly dysregulated. Of these, six were validated in tissue samples, but only miR-126 and miR-142-3p were elevated in plasma in HBV patients with HCC compared to patients without HCC. Neither performed better than AFP alone, but the combination of either microRNA with AFP improved the AUC to 0.92. No difference in miR-126 levels in patients with HCV-related HCC or non-viral HCC was detected, suggesting that differences in the underlying etiology of HCC may affect the predictive power of microRNA biomarkers. While HBV is the most common cause of HCC in Asia and other HBV-endemic regions, HCV-related HCC is more common in Japan, and the world-wide incidence of HBV is gradually declining due to vaccination, whereas incidence of non-viral, non-alcoholic HCC due to non-alcoholic steatohepatitis and other causes is increasing [74]. Therefore, biomarkers developed and validated in one region should be validated in regions in with different underlying etiologies.

The predictive effects of various microRNAs in combination have been evaluated in a number of studies, although so far there has been little overlap among panels, partly due to differences in the source and measurement of microRNAs, making comparison difficult. The combination of miR-126 and miR-141 measured in tumor samples was reported to be discriminative between HCC and metastatic adenocarcinoma of the liver, making them potentially useful diagnostic markers for tissue samples [116]. Using serum samples, Li *et al.* [87] reported that the combination of miR-25, mR-375, and let-7f could discriminate HBV-associated HCC samples from healthy controls, whereas the combination of miR-16, miR-195, and miR-199a could discriminate HBV-associated HCC from chronic HBV infection. In a retrospective study, Liu *et al.* [23] reported that miR-15b, miR-21, miR-130b, and miR-183 were up-regulated in HCC tumor tissue relative to adjacent non-tumor tissue. Levels of these microRNAs were detectable in serum and cell culture supernatant, and serum levels declined significantly following surgical resection. In a validation study, they found that the combination of miR-15b and miR-130b was strongly predictive of HCC (AUC 0.98) and could detect HCC earlier than AFP [23]. The combination of miR-15b and miR-130 could also discriminate HCC from healthy samples with high sensitivity and specificity. Similarly, Lin *et al.* [117] identified a set of 19 microRNAs that were up-regulated in serum of patients with HBV-related HCC compared to patients with chronic HBV. Using a training cohort and two independent validation cohorts, they developed and validated a seven-microRNA panel including miR-29a, miR-29c, miR-133a, miR-143, miR-145, miR-192, and miR-505. This panel was more sensitive than AFP and could detect small AFP-negative HCC samples, providing hope for earlier detection of HCC. While microRNAs have great potential as a biomarker for HCC, there is no consensus yet on optimal microRNA panels or detection methods [7]. A potential limitation of studies to date involves the initial screening of candidate microRNAs using microRNA microarrays, which typically contain only a limited set of probes that may not include recently-identified microRNAs nor adequately prevent cross-hybridization with unrepresented microRNAs [57]. The increasing use of global transcriptome sequencing should offer a more unbiased approach to identification of candidate RNAs.

In addition to their potential roles in diagnosis and classification, microRNAs might also provide a way to monitor response to therapy as well as serve as drug targets. For example, low tumor miR-26 levels is associated with better response to interferon therapy, but is also associated with poorer survival [118], and miR-29a-5p is associated with early post-resection recurrence of HCC in patients with HBV [119]. MicroRNAs themselves might also serve as therapeutic agents. In an interesting study, Kourtidis *et al.* [120] note that microRNAs such as miR-30b normally suppress cell growth when cells come into contact but lose control when adhesion is disrupted in cancer. They showed that, while adhesion proteins normally interact with components of the microprocessor complex in the cytoplasm, this structure was lost in most of the tumors they examined. However, restoration of miR-30b levels reversed the abnormal cell growth. While targeted delivery and expression of therapeutic microRNAs will likely present a challenge, greater understanding of the roles of microRNAs and other non-coding RNAs may lead to more effective and better tolerated treatments for HCC and other cancers.

## 11. Mechanisms of MicroRNA Dysregulation

MicroRNAs are often located at fragile sites and breakpoint regions associated with cancer development [121]. Changes in microRNA expression in HCC partially reflects underlying genomic instability affecting microRNA gene expression through mutations, translocations, copy number changes, deletions, DNA methylation, and histone modifications [11,122,123,124]. For example, histone deacetylases, which regulate gene expression through chromatin remodeling, are frequently up-regulated in HCC which, in turn, reduces expression of miR-449, a c-Met inhibitor [124]. Increased c-Met expression prevents apoptosis and promotes cell proliferation. He *et al.* [125] reported that miR-191 expression is 59% higher in HCC tumor *versus* adjacent non-tumor tissue and is associated with poor prognosis. Using methylation-specific PCR and bisulfite sequencing PCR, they showed that hypomethylation was correlated with elevated miR-191 expression levels, which induced a mesenchyme-like transition characterized by loss of adhesion, change from epithelial to mesenchymal cell markers, and increased invasiveness, whereas down-regulation of miR-191 had the opposite effect. Understanding the mechanism behind microRNA dysregulation is not necessary to develop biomarkers, but elucidating the cellular and metabolic changes underlying disruption of microRNA expression patterns might provide greater insight into pathogenesis and reveal potential therapeutic targets.

## 12. Conclusions

Development of microRNA panels, either alone or in combination with classical biomarkers, might eventually be used to classify samples with respect to liver function and progression to HCC, helping to inform treatment decisions and establish prognosis. However, several obstacles remain, including standardization of quantification protocols and validation of microRNA panels. Differences in microRNA expression should be examined with respect to age, sex, genetic background, and underlying etiology, and potential mis-annotation or cross-hybridization of potential microRNA biomarkers should be confirmed. Use of global RNA sequencing to identify candidate microRNAs and selection of appropriate internal controls should improve reproducibility of results among studies and overcome limitations inherent in earlier microarray studies. Monitoring of changes in microRNA profiles might provide earlier warning of changes in liver function preceding appearance of HCC, resulting in more effective treatment and improved survival. Such improvements are urgently needed, especially among the aging chronic HBV patient population.

## References

[1] Jemal A., Bray F., Center M.M., Ferlay J., Ward E., Forman D. (2011). Global cancer statistics. CA Cancer J. Clin..

[2] Fattovich G., Stroffolini T., Zagni I., Donato F. (2004). Hepatocellular carcinoma in cirrhosis: Incidence and risk factors. Gastroenterology.

[3] Parkin D.M., Bray F., Ferlay J., Pisani P. (2005). Global cancer statistics, 2002. CA Cancer J. Clin..

[4] Aleman S., Rahbin N., Weiland O., Davidsdottir L., Hedenstierna M., Rose N., Verbaan H., Stal P., Carlsson T., Norrgren H. (2013). A risk for hepatocellular carcinoma persists long-term after sustained virologic response in patients with hepatitis C-associated liver cirrhosis. Clin. Infect. Dis..

[5] Turchinovich A., Weiz L., Langheinz A., Burwinkel B. (2011). Characterization of extracellular circulating microrna. Nucleic Acids Res..

[6] Lewis B.P., Burge C.B., Bartel D.P. (2005). Conserved seed pairing, often flanked by adenosines, indicates that thousands of human genes are microRNA targets. Cell.

[7] Anwar S.L., Lehmann U. (2015). Micrornas: Emerging novel clinical biomarkers for hepatocellular carcinomas. J. Clin. Med..

[8] Kerr T.A., Korenblat K.M., Davidson N.O. (2011). MicroRNAs and liver disease. Transl. Res..

[9] Li H., Yang R., Fan X., Gu T., Zhao Z., Chang D., Wang W., Wang C. (2012). MicroRNA array analysis of microRNAs related to systemic scleroderma. Rheumatol. Int..

[10] Te J.L., Dozmorov I.M., Guthridge J.M., Nguyen K.L., Cavett J.W., Kelly J.A., Bruner G.R., Harley J.B., Ojwang J.O. (2010). Identification of unique microRNA signature associated with lupus nephritis. PLoS ONE.

[11] Croce C.M., Calin G.A. (2005). MiRNAs, cancer, and stem cell division. Cell.

[12] Calin G.A., Dumitru C.D., Shimizu M., Bichi R., Zupo S., Noch E., Aldler H., Rattan S., Keating M., Rai K. (2002). Frequent deletions and down-regulation of micro-RNA genes miR15 and miR16 at 13q14 in chronic lymphocytic leukemia. Proc. Natl. Acad. Sci. USA.

[13] Cai X., Hagedorn C.H., Cullen B.R. (2004). Human microRNAs are processed from capped, polyadenylated transcripts that can also function as mRNAs. RNA.

[14] Lawrie C.H., Gal S., Dunlop H.M., Pushkaran B., Liggins A.P., Pulford K., Banham A.H., Pezzella F., Boultwood J., Wainscoat J.S. (2008). Detection of elevated levels of tumour-associated microRNAs in serum of patients with diffuse large B-cell lymphoma. Br. J. Haematol..

[15] Liu A.M., Zhang C., Burchard J., Fan S.T., Wong K.F., Dai H., Poon R.T., Luk J.M. (2011). Global regulation on microRNA in hepatitis B virus-associated hepatocellular carcinoma. Omics.

[16] Bala S., Marcos M., Szabo G. (2009). Emerging role of microRNAs in liver diseases. World J. Gastroenterol..

[17] Ji F., Yang B., Peng X., Ding H., You H., Tien P. (2011). Circulating microRNAs in hepatitis B virus-infected patients. J. Viral Hepat..

[18] Mitchell P.S., Parkin R.K., Kroh E.M., Fritz B.R., Wyman S.K., Pogosova-Agadjanyan E.L., Peterson A., Noteboom J., O’Briant K.C., Allen A. (2008). Circulating microRNAs as stable blood-based markers for cancer detection. Proc. Natl. Acad. Sci. USA.

[19] Ura S., Honda M., Yamashita T., Ueda T., Takatori H., Nishino R., Sunakozaka H., Sakai Y., Horimoto K., Kaneko S. (2009). Differential microRNA expression between hepatitis B and hepatitis C leading disease progression to hepatocellular carcinoma. Hepatology.

[20] Hayes C.N., Akamatsu S., Tsuge M., Miki D., Akiyama R., Abe H., Ochi H., Hiraga N., Imamura M., Takahashi S. (2012). Hepatitis B virus-specific miRNAs and argonaute2 play a role in the viral life cycle. PLoS ONE.

[21] Shwetha S., Gouthamchandra K., Chandra M., Ravishankar B., Khaja M.N., Das S. (2013). Circulating miRNA profile in HCV infected serum: Novel insight into pathogenesis. Sci. Rep..

[22] Novellino L., Rossi R.L., Bonino F., Cavallone D., Abrignani S., Pagani M., Brunetto M.R. (2012). Circulating hepatitis b surface antigen particles carry hepatocellular microRNAs. PLoS ONE.

[23] Liu A.M., Yao T.J., Wang W., Wong K.F., Lee N.P., Fan S.T., Poon R.T., Gao C., Luk J.M. (2012). Circulating miR-15b and miR-130b in serum as potential markers for detecting hepatocellular carcinoma: A retrospective cohort study. BMJ Open.

[24] Cortez M.A., Bueso-Ramos C., Ferdin J., Lopez-Berestein G., Sood A.K., Calin G.A. (2011). MicroRNAs in body fluids—The mix of hormones and biomarkers. Nat. Rev. Clin. Oncol..

[25] Arroyo J.D., Chevillet J.R., Kroh E.M., Ruf I.K., Pritchard C.C., Gibson D.F., Mitchell P.S., Bennett C.F., Pogosova-Agadjanyan E.L., Stirewalt D.L. (2011). Argonaute2 complexes carry a population of circulating microRNAs independent of vesicles in human plasma. Proc. Natl. Acad. Sci. USA.

[26] Gallo A., Tandon M., Alevizos I., Illei G.G. (2012). The majority of microRNAs detectable in serum and saliva is concentrated in exosomes. PLoS ONE.

[27] Huang X., Yuan T., Tschannen M., Sun Z., Jacob H., Du M., Liang M., Dittmar R.L., Liu Y., Kohli M. (2013). Characterization of human plasma-derived exosomal RNAs by deep sequencing. BMC Genom..

[28] Conde-Vancells J., Rodriguez-Suarez E., Embade N., Gil D., Matthiesen R., Valle M., Elortza F., Lu S.C., Mato J.M., Falcon-Perez J.M. (2008). Characterization and comprehensive proteome profiling of exosomes secreted by hepatocytes. J. Proteome Res..

[29] Li J., Liu K., Liu Y., Xu Y., Zhang F., Yang H., Liu J., Pan T., Chen J., Wu M. (2013). Exosomes mediate the cell-to-cell transmission of IFN-α-induced antiviral activity. Nat. Immunol..

[30] Taylor C.J., Satoor S.N., Ranjan A.K., Pereira e Cotta M.V., Joglekar M.V. (2012). A protocol for measurement of noncoding RNA in human serum. Exp. Diabetes Res..

[31] Natarajan S.K., Pachunka J.M., Mott J.L. (2015). Role of microRNAs in alcohol-induced multi-organ injury. Biomolecules.

[32] Marabita F., de Candia P., Torri A., Tegner J., Abrignani S., Rossi R.L. (2015). Normalization of circulating microRNA expression data obtained by quantitative real-time RT-PCR. Brief. Bioinform..

[33] Benz F., Roderburg C., Vargas Cardenas D., Vucur M., Gautheron J., Koch A., Zimmermann H., Janssen J., Nieuwenhuijsen L., Luedde M. (2013). U6 is unsuitable for normalization of serum miRNA levels in patients with sepsis or liver fibrosis. Exp. Mol. Med..

[34] McDonald J.S., Milosevic D., Reddi H.V., Grebe S.K., Algeciras-Schimnich A. (2011). Analysis of circulating microRNA: Preanalytical and analytical challenges. Clin. Chem..

[35] Li L.Z., Zhang C.Z., Liu L.L., Yi C., Lu S.X., Zhou X., Zhang Z.J., Peng Y.H., Yang Y.Z., Yun J.P. (2014). MiR-720 inhibits tumor invasion and migration in breast cancer by targeting twist1. Carcinogenesis.

[36] Schopman N.C., Heynen S., Haasnoot J., Berkhout B. (2010). A miRNA-tRNA mix-up: TRNA origin of proposed miRNA. RNA Biol..

[37] Langenberger D., Bartschat S., Hertel J., Hoffmann S., Tafer H., Stadler P., Norberto de Souza O., Telles G., Palakal M. (2011). MicroRNA or not microRNA?. Advances in Bioinformatics and Computational Biology.

[38] Dhahbi J.M., Spindler S.R., Atamna H., Yamakawa A., Boffelli D., Mote P., Martin D.I. (2013). 5′ tRNA halves are present as abundant complexes in serum, concentrated in blood cells, and modulated by aging and calorie restriction. BMC Genom..

[39] Anderson P., Ivanov P. (2014). TRNA fragments in human health and disease. FEBS Lett..

[40] Selitsky S.R., Baran-Gale J., Honda M., Yamane D., Masaki T., Fannin E.E., Guerra B., Shirasaki T., Shimakami T., Kaneko S. (2015). Small tRNA-derived RNAs are increased and more abundant than microRNAs in chronic hepatitis B and C. Sci. Rep..

[41] Law P.T., Qin H., Ching A.K., Lai K.P., Co N.N., He M., Lung R.W., Chan A.W., Chan T.F., Wong N. (2013). Deep sequencing of small RNA transcriptome reveals novel non-coding RNAs in hepatocellular carcinoma. J. Hepatol..

[42] Hou J., Lin L., Zhou W., Wang Z., Ding G., Dong Q., Qin L., Wu X., Zheng Y., Yang Y. (2011). Identification of miRNomes in human liver and hepatocellular carcinoma reveals miR-199a/b-3p as therapeutic target for hepatocellular carcinoma. Cancer Cell.

[43] Gamazon E.R., Innocenti F., Wei R., Wang L., Zhang M., Mirkov S., Ramirez J., Huang R.S., Cox N.J., Ratain M.J. (2013). A genome-wide integrative study of microRNAs in human liver. BMC Genom..

[44] Jopling C. (2012). Liver-specific microRNA-122: Biogenesis and function. RNA Biol..

[45] Sharma S., Eghbali M. (2014). Influence of sex differences on microRNA gene regulation in disease. Biol. Sex Differ..

[46] Roderburg C., Urban G.W., Bettermann K., Vucur M., Zimmermann H., Schmidt S., Janssen J., Koppe C., Knolle P., Castoldi M. (2011). Micro-RNA profiling reveals a role for miR-29 in human and murine liver fibrosis. Hepatology.

[47] Migita K., Komori A., Kozuru H., Jiuchi Y., Nakamura M., Yasunami M., Furukawa H., Abiru S., Yamasaki K., Nagaoka S. (2015). Circulating microRNA profiles in patients with type-1 autoimmune hepatitis. PLoS ONE.

[48] Tan Y., Ge G., Pan T., Wen D., Gan J. (2014). A pilot study of serum microRNAs panel as potential biomarkers for diagnosis of nonalcoholic fatty liver disease. PLoS ONE.

[49] Li L.M., Wang D., Zen K. (2014). MicroRNAs in drug-induced liver injury. J. Clin. Transl. Hepatol..

[50] Bala S., Petrasek J., Mundkur S., Catalano D., Levin I., Ward J., Alao H., Kodys K., Szabo G. (2012). Circulating microRNAs in exosomes indicate hepatocyte injury and inflammation in alcoholic, drug-induced, and inflammatory liver diseases. Hepatology.

[51] Wang K., Zhang S., Marzolf B., Troisch P., Brightman A., Hu Z., Hood L.E., Galas D.J. (2009). Circulating microRNAs, potential biomarkers for drug-induced liver injury. Proc. Natl. Acad. Sci. USA.

[52] Fukushima T., Hamada Y., Yamada H., Horii I. (2007). Changes of micro-RNA expression in rat liver treated by acetaminophen or carbon tetrachloride—Regulating role of micro-RNA for RNA expression. J. Toxicol. Sci..

[53] Starkey Lewis P.J., Dear J., Platt V., Simpson K.J., Craig D.G., Antoine D.J., French N.S., Dhaun N., Webb D.J., Costello E.M. (2011). Circulating microRNAs as potential markers of human drug-induced liver injury. Hepatology.

[54] Ding X., Ding J., Ning J., Yi F., Chen J., Zhao D., Zheng J., Liang Z., Hu Z., Du Q. (2012). Circulating microRNA-122 as a potential biomarker for liver injury. Mol. Med. Rep..

[55] Dolganiuc A., Petrasek J., Kodys K., Catalano D., Mandrekar P., Velayudham A., Szabo G. (2009). MicroRNA expression profile in lieber-decarli diet-induced alcoholic and methionine choline deficient diet-induced nonalcoholic steatohepatitis models in mice. Alcohol. Clin. Exp. Res..

[56] Chen Y.P., Jin X., Xiang Z., Chen S.H., Li Y.M. (2013). Circulating microRNAs as potential biomarkers for alcoholic steatohepatitis. Liver Int..

[57] Baffy G. (2015). MicroRNAs in nonalcoholic fatty liver disease. J. Clin. Med..

[58] Zarfeshani A., Ngo S., Sheppard A.M. (2015). MicroRNA expression relating to dietary-induced liver steatosis and nash. J. Clin. Med..

[59] Jin X., Ye Y.F., Chen S.H., Yu C.H., Liu J., Li Y.M. (2009). MicroRNA expression pattern in different stages of nonalcoholic fatty liver disease. Dig. Liver Dis..

[60] Li S., Chen X., Zhang H., Liang X., Xiang Y., Yu C., Zen K., Li Y., Zhang C.Y. (2009). Differential expression of microRNAs in mouse liver under aberrant energy metabolic status. J. Lipid Res..

[61] Feng Y.Y., Xu X.Q., Ji C.B., Shi C.M., Guo X.R., Fu J.F. (2014). Aberrant hepatic microRNA expression in nonalcoholic fatty liver disease. Cell. Physiol. Biochem..

[62] Cheung O., Puri P., Eicken C., Contos M.J., Mirshahi F., Maher J.W., Kellum J.M., Min H., Luketic V.A., Sanyal A.J. (2008). Nonalcoholic steatohepatitis is associated with altered hepatic microRNA expression. Hepatology.

[63] Cermelli S., Ruggieri A., Marrero J.A., Ioannou G.N., Beretta L. (2011). Circulating microRNAs in patients with chronic hepatitis c and non-alcoholic fatty liver disease. PLoS ONE.

[64] Pirola C.J., Fernandez Gianotti T., Castano G.O., Mallardi P., San Martino J., Mora Gonzalez Lopez Ledesma M., Flichman D., Mirshahi F., Sanyal A.J., Sookoian S. (2015). Circulating microRNA signature in non-alcoholic fatty liver disease: From serum non-coding RNAs to liver histology and disease pathogenesis. Gut.

[65] Teng K.Y., Ghoshal K. (2015). Role of noncoding RNAs as biomarker and therapeutic targets for liver fibrosis. Gene Expr..

[66] Friedman S.L., Roll F.J., Boyles J., Bissell D.M. (1985). Hepatic lipocytes: The principal collagen-producing cells of normal rat liver. Proc. Natl. Acad. Sci. USA.

[67] Bataller R., Brenner D.A. (2005). Liver fibrosis. J. Clin. Investig..

[68] Zhang Y., Wu L., Wang Y., Zhang M., Li L., Zhu D., Li X., Gu H., Zhang C.Y., Zen K. (2012). Protective role of estrogen-induced miRNA-29 expression in carbon tetrachloride-induced mouse liver injury. J. Biol. Chem..

[69] Peng X., Li Y., Walters K.A., Rosenzweig E.R., Lederer S.L., Aicher L.D., Proll S., Katze M.G. (2009). Computational identification of hepatitis C virus associated microRNA-mRNA regulatory modules in human livers. BMC Genom..

[70] Zhang G.L., Li Y.X., Zheng S.Q., Liu M., Li X., Tang H. (2010). Suppression of hepatitis b virus replication by microRNA-199a-3p and microRNA-210. Antivir. Res..

[71] Potenza N., Papa U., Mosca N., Zerbini F., Nobile V., Russo A. (2011). Human microRNA hsa-miR-125a-5p interferes with expression of hepatitis B virus surface antigen. Nucleic Acids Res..

[72] Wu F.L., Jin W.B., Li J.H., Guo A.G. (2011). Targets for human encoded microRNAs in hbv genes. Virus Genes.

[73] Kohno T., Tsuge M., Murakami E., Hiraga N., Abe H., Miki D., Imamura M., Ochi H., Hayes C.N., Chayama K. (2014). Human microRNA hsa-miR-1231 suppresses hepatitis B virus replication by targeting core mRNA. J. Viral Hepat..

[74] Chen Y., Shen A., Rider P.J., Yu Y., Wu K., Mu Y., Hao Q., Liu Y., Gong H., Zhu Y. (2011). A liver-specific microRNA binds to a highly conserved RNA sequence of hepatitis B virus and negatively regulates viral gene expression and replication. FASEB J..

[75] Wang S., Qiu L., Yan X., Jin W., Wang Y., Chen L., Wu E., Ye X., Gao G.F., Wang F. (2012). Loss of miR-122 expression in patients with hepatitis B enhances hepatitis B virus replication through cyclin G1 modulated P53 activity. Hepatology.

[76] Hu J., Xu Y., Hao J., Wang S., Li C., Meng S. (2012). MiR-122 in hepatic function and liver diseases. Protein Cell.

[77] Chang J., Nicolas E., Marks D., Sander C., Lerro A., Buendia M.A., Xu C., Mason W.S., Moloshok T., Bort R. (2004). MiR-122, a mammalian liver-specific microRNA, is processed from hcr mRNA and may downregulate the high affinity cationic amino acid transporter CAT-1. RNA Biol..

[78] Qiu L., Fan H., Jin W., Zhao B., Wang Y., Ju Y., Chen L., Chen Y., Duan Z., Meng S. (2010). MiR-122-induced down-regulation of HO-1 negatively affects miR-122-mediated suppression of HBV. Biochem. Biophys. Res. Commun..

[79] Li D., Liu X., Lin L., Hou J., Li N., Wang C., Wang P., Zhang Q., Zhang P., Zhou W. (2011). MicroRNA-99a inhibits hepatocellular carcinoma growth and correlates with prognosis of patients with hepatocellular carcinoma. J. Biol. Chem..

[80] Takata A., Otsuka M., Kojima K., Yoshikawa T., Kishikawa T., Yoshida H., Koike K. (2011). MicroRNA-22 and microRNA-140 suppress NF-κB activity by regulating the expression of NF-κB coactivators. Biochem. Biophys. Res. Commun..

[81] Xu D., Takeshita F., Hino Y., Fukunaga S., Kudo Y., Tamaki A., Matsunaga J., Takahashi R.U., Takata T., Shimamoto A. (2011). MiR-22 represses cancer progression by inducing cellular senescence. J. Cell Biol..

[82] Shi C., Xu X. (2013). MicroRNA-22 is down-regulated in hepatitis B virus-related hepatocellular carcinoma. Biomed. Pharmacother..

[83] Hu W., Wang X., Ding X., Li Y., Zhang X., Xie P., Yang J., Wang S. (2012). MicroRNA-141 represses HBV replication by targeting PPARA. PLoS ONE.

[84] Zhang X., Zhang E., Ma Z., Pei R., Jiang M., Schlaak J.F., Roggendorf M., Lu M. (2011). Modulation of hepatitis b virus replication and hepatocyte differentiation by microRNA-1. Hepatology.

[85] Jin J., Tang S., Xia L., Du R., Xie H., Song J., Fan R., Bi Q., Chen Z., Yang G. (2013). MicroRNA-501 promotes hbv replication by targeting HBXIP. Biochem. Biophys. Res. Commun..

[86] Jin W.B., Wu F.L., Kong D., Guo A.G. (2007). Hbv-encoded microRNA candidate and its target. Comput. Biol. Chem..

[87] Li L.M., Hu Z.B., Zhou Z.X., Chen X., Liu F.Y., Zhang J.F., Shen H.B., Zhang C.Y., Zen K. (2010). Serum microRNA profiles serve as novel biomarkers for HBV infection and diagnosis of HBV-positive hepatocarcinoma. Cancer Res..

[88] Arataki K., Hayes C.N., Akamatsu S., Akiyama R., Abe H., Tsuge M., Miki D., Ochi H., Hiraga N., Imamura M. (2013). Circulating microRNA-22 correlates with microRNA-122 and represents viral replication and liver injury in patients with chronic hepatitis B. J. Med. Virol..

[89] Akamatsu S., Hayes C.N., Tsuge M., Miki D., Akiyama R., Abe H., Ochi H., Hiraga N., Imamura M., Takahashi S. (2015). Differences in serum microRNA profiles in hepatitis B and C virus infection. J. Infect..

[90] Giray B.G., Emekdas G., Tezcan S., Ulger M., Serin M.S., Sezgin O., Altintas E., Tiftik E.N. (2014). Profiles of serum microRNAs; miR-125b-5p and miR223–3p serve as novel biomarkers for HBV-positive hepatocellular carcinoma. Mol. Biol. Rep..

[91] Ren M., Qin D., Li K., Qu J., Wang L., Wang Z., Huang A., Tang H. (2012). Correlation between hepatitis B virus protein and microRNA processor drosha in cells expressing HBV. Antivir. Res..

[92] Steuerwald N.M., Parsons J.C., Bennett K., Bates T.C., Bonkovsky H.L. (2010). Parallel microRNA and mRNA expression profiling of (genotype 1b) human hepatoma cells expressing hepatitis C virus. Liver Int..

[93] Pedersen I.M., Cheng G., Wieland S., Volinia S., Croce C.M., Chisari F.V., David M. (2007). Interferon modulation of cellular microRNAs as an antiviral mechanism. Nature.

[94] Jopling C.L., Yi M., Lancaster A.M., Lemon S.M., Sarnow P. (2005). Modulation of hepatitis C virus RNA abundance by a liver-specific microRNA. Science.

[95] Lanford R.E., Hildebrandt-Eriksen E.S., Petri A., Persson R., Lindow M., Munk M.E., Kauppinen S., Orum H. (2010). Therapeutic silencing of microRNA-122 in primates with chronic hepatitis C virus infection. Science.

[96] Banaudha K., Kaliszewski M., Korolnek T., Florea L., Yeung M.L., Jeang K.T., Kumar A. (2011). MicroRNA silencing of tumor suppressor DLC-1 promotes efficient hepatitis C virus replication in primary human hepatocytes. Hepatology.

[97] Ishida H., Tatsumi T., Hosui A., Nawa T., Kodama T., Shimizu S., Hikita H., Hiramatsu N., Kanto T., Hayashi N. (2011). Alterations in microRNA expression profile in HCV-infected hepatoma cells: Involvement of miR-491 in regulation of HCV replication via the PI3 kinase/akt pathway. Biochem. Biophys. Res. Commun..

[98] Murakami Y., Aly H.H., Tajima A., Inoue I., Shimotohno K. (2009). Regulation of the hepatitis C virus genome replication by miR-199a. J. Hepatol..

[99] Hou W., Tian Q., Zheng J., Bonkovsky H.L. (2010). MicroRNA-196 represses Bach1 protein and hepatitis C virus gene expression in human hepatoma cells expressing hepatitis C viral proteins. Hepatology.

[100] Bandyopadhyay S., Friedman R.C., Marquez R.T., Keck K., Kong B., Icardi M.S., Brown K.E., Burge C.B., Schmidt W.N., Wang Y. (2011). Hepatitis C virus infection and hepatic stellate cell activation downregulate miR-29: MiR-29 overexpression reduces hepatitis C viral abundance in culture. J. Infect. Dis..

[101] Cheng J.C., Yeh Y.J., Tseng C.P., Hsu S.D., Chang Y.L., Sakamoto N., Huang H.D. (2012). Let-7b is a novel regulator of hepatitis C virus replication. Cell. Mol. Life Sci..

[102] Bhanja Chowdhury J., Shrivastava S., Steele R., Di Bisceglie A.M., Ray R., Ray R.B. (2012). Hepatitis C virus infection modulates expression of interferon stimulatory gene *IFITM1* by upregulating miR-130a. J. Virol..

[103] Shirasaki T., Honda M., Shimakami T., Horii R., Yamashita T., Sakai Y., Sakai A., Okada H., Watanabe R., Murakami S. (2013). MicroRNA-27a regulates lipid metabolism and inhibits hepatitis C virus replication in human hepatoma cells. J. Virol..

[104] You J.S., Jones P.A. (2012). Cancer genetics and epigenetics: Two sides of the same coin?. Cancer Cell.

[105] Ashworth A., Lord C.J., Reis-Filho J.S. (2011). Genetic interactions in cancer progression and treatment. Cell.

[106] Pogribny I.P., Rusyn I. (2014). Role of epigenetic aberrations in the development and progression of human hepatocellular carcinoma. Cancer Lett..

[107] Bertino G., Ardiri A.M., Calvagno G.S., Bertino N., Boemi P.M. (2010). Prognostic and diagnostic value of des-γ-carboxy prothrombin in liver cancer. Drug News Perspect..

[108] Durazo F.A., Blatt L.M., Corey W.G., Lin J.H., Han S., Saab S., Busuttil R.W., Tong M.J. (2008). Des-γ-carboxyprothrombin, α-fetoprotein and AFP-L3 in patients with chronic hepatitis, cirrhosis and hepatocellular carcinoma. J. Gastroenterol. Hepatol..

[109] Borel F., Konstantinova P., Jansen P.L. (2012). Diagnostic and therapeutic potential of miRNA signatures in patients with hepatocellular carcinoma. J. Hepatol..

[110] Ladeiro Y., Couchy G., Balabaud C., Bioulac-Sage P., Pelletier L., Rebouissou S., Zucman-Rossi J. (2008). MicroRNA profiling in hepatocellular tumors is associated with clinical features and oncogene/tumor suppressor gene mutations. Hepatology.

[111] Huang S., He X. (2011). The role of microRNAs in liver cancer progression. Br. J. Cancer.

[112] Qi P., Cheng S.Q., Wang H., Li N., Chen Y.F., Gao C.F. (2011). Serum microRNAs as biomarkers for hepatocellular carcinoma in chinese patients with chronic hepatitis B virus infection. PLoS ONE.

[113] Wang Y., Lee A.T., Ma J.Z., Wang J., Ren J., Yang Y., Tantoso E., Li K.B., Ooi L.L., Tan P. (2008). Profiling microRNA expression in hepatocellular carcinoma reveals microRNA-224 up-regulation and apoptosis inhibitor-5 as a microRNA-224-specific target. J. Biol. Chem..

[114] Zhuang L.P., Meng Z.Q. (2015). Serum miR-224 reflects stage of hepatocellular carcinoma and predicts survival. Biomed. Res. Int..

[115] Ghosh A., Ghosh A., Datta S., Dasgupta D., Das S., Ray S., Gupta S., Datta S., Chowdhury A., Chatterjee R. (2016). Hepatic miR-126 is a potential plasma biomarker for detection of hepatitis B virus infected hepatocellular carcinoma. Int. J. Cancer.

[116] Barshack I., Meiri E., Rosenwald S., Lebanony D., Bronfeld M., Aviel-Ronen S., Rosenblatt K., Polak-Charcon S., Leizerman I., Ezagouri M. (2010). Differential diagnosis of hepatocellular carcinoma from metastatic tumors in the liver using microRNA expression. Int. J. Biochem. Cell Biol..

[117] Lin X.J., Chong Y., Guo Z.W., Xie C., Yang X.J., Zhang Q., Li S.P., Xiong Y., Yuan Y., Min J. (2015). A serum microRNA classifier for early detection of hepatocellular carcinoma: A multicentre, retrospective, longitudinal biomarker identification study with a nested case-control study. Lancet Oncol..

[118] Ji J., Shi J., Budhu A., Yu Z., Forgues M., Roessler S., Ambs S., Chen Y., Meltzer P.S., Croce C.M. (2009). MicroRNA expression, survival, and response to interferon in liver cancer. N. Engl. J. Med..

[119] Zhu H.T., Dong Q.Z., Sheng Y.Y., Wei J.W., Wang G., Zhou H.J., Ren N., Jia H.L., Ye Q.H., Qin L.X. (2012). MicroRNA-29a-5p is a novel predictor for early recurrence of hepatitis b virus-related hepatocellular carcinoma after surgical resection. PLoS ONE.

[120] Kourtidis A., Ngok S.P., Pulimeno P., Feathers R.W., Carpio L.R., Baker T.R., Carr J.M., Yan I.K., Borges S., Perez E.A. (2015). Distinct E-cadherin-based complexes regulate cell behaviour through miRNA processing or Src and p120 catenin activity. Nat. Cell Biol..

[121] Calin G.A., Sevignani C., Dumitru C.D., Hyslop T., Noch E., Yendamuri S., Shimizu M., Rattan S., Bullrich F., Negrini M. (2004). Human microRNA genes are frequently located at fragile sites and genomic regions involved in cancers. Proc. Natl. Acad. Sci. USA.

[122] Anwar S.L., Albat C., Krech T., Hasemeier B., Schipper E., Schweitzer N., Vogel A., Kreipe H., Lehmann U. (2013). Concordant hypermethylation of intergenic microRNA genes in human hepatocellular carcinoma as new diagnostic and prognostic marker. Int. J. Cancer.

[123] Anwar S.L., Lehmann U. (2014). DNA methylation, microRNAs, and their crosstalk as potential biomarkers in hepatocellular carcinoma. World J. Gastroenterol..

[124] Buurman R., Gurlevik E., Schaffer V., Eilers M., Sandbothe M., Kreipe H., Wilkens L., Schlegelberger B., Kuhnel F., Skawran B. (2012). Histone deacetylases activate hepatocyte growth factor signaling by repressing microRNA-449 in hepatocellular carcinoma cells. Gastroenterology.

[125] He Y., Cui Y., Wang W., Gu J., Guo S., Ma K., Luo X. (2011). Hypomethylation of the hsa-miR-191 locus causes high expression of hsa-miR-191 and promotes the epithelial-to-mesenchymal transition in hepatocellular carcinoma. Neoplasia.

